# Editorial: Case reports in Schizophrenia and psychotic disorders: 2023

**DOI:** 10.3389/fpsyt.2025.1623129

**Published:** 2025-06-05

**Authors:** Massimo Tusconi, Gabriele Nibbio, Rishab Gupta, Erika Carr

**Affiliations:** ^1^ Department of Medical Sciences and Public Health, University of Cagliari, Cagliari, Italy; ^2^ Department of Clinical and Experimental Sciences, University of Brescia, Brescia, Italy; ^3^ Brigham and Women’s Faulkner Hospital, Harvard Medical School, Boston, MA, United States; ^4^ Department of Psychiatry, Yale University School of Medicine, New Haven, CT, United States

**Keywords:** case reports, schizophrenia, psychosis, Catatonia, encephalitis, treatment resistant psychosis

Case reports have long played a fundamental role in the evolution of medical knowledge (1–3), offering food for thought on rare or complex clinical conditions that could hardly be adequately evaluated in large-scale studies (4). In the context of Schizophrenia and psychotic disorders, the importance of meticulously documented case reports cannot be underestimated (5, 6). These clinical descriptions, although singular (7–9) in their scope, often catalyze new lines of investigation, challenge prevailing paradigms (10–12), focus on aspects that have only been marginally explored, and deepen our understanding of the clinical, neurobiological, and psychosocial dimensions of psychosis (13–15). Schizophrenia and related psychotic disorders remain among the most enigmatic conditions in psychiatry (16, 17), often associated with cognitive impairment, poor real-world functioning and high levels of disability (7). Despite significant advances in neuroimaging, genetics, and psychopharmacology, their etiopathogenesis is only partially understood (18). Clinical heterogeneity, diagnostic complexity, and variable responses to treatment further emphasize the need for in-depth clinical observations (19–21). Case reports offer a powerful means of exploring these complexities, often revealing atypical symptom patterns and comorbid conditions, new therapeutic responses, and the profound interaction between individual biology and environment (22–25). In recent years, the psychiatric community has witnessed a revival of appreciation for qualitative and phenomenological approaches, particularly in the study of psychotic disorders (26–29). Through case reports, research can highlight the nuanced experience of psychosis as lived by the patient, illuminating dimensions of suffering, healing, insight, and meaning that standardized measures may fail to capture, regardless of inclusion criteria (17, 30, 31). Such narratives humanize psychiatric illness, enrich clinical empathy, and promote a more patient-centered approach to care (32, 33) of particular importance in working with those who experience psychosis and stigma.

Furthermore, case reports have a unique pedagogical value (34). For medical students, trainee doctors, and doctors early in their careers, engaging in preparing and publishing a case report cultivates essential skills in clinical reasoning, literature synthesis, and scientific communication (35, 36). In the field of psychosis, where patients’ journeys often defy the most classic textbook definitions, learning through real-world clinical narratives can be particularly formative (37, 38). This Research Topic, Case Reports in Schizophrenia and Psychotic Disorders, aims to highlight the breadth and depth of contemporary clinical psychiatry by describing reports that show diagnostic dilemmas, rare comorbidities, new therapeutic approaches or ethical challenges in the treatment of individuals with psychosis, and by delving deeper with contributions that incorporate multidisciplinary perspectives, drawing from neurology, pharmacology, psychology, and social work.

The issue of managing the macroscopic aspects of Schizophrenia is considered by Takada et al. in their case report on the long-term management of occlusion after surgical-orthodontic treatment for a patient with drug-induced open bite developed after the onset of Schizophrenia; in this case, the synergistic effect of the use of a removable orthodontic appliance and medication management to achieve psychological and occlusal stability is reported, emphasizing how medication control was considered essential to improve the patient’s drug-induced open bite and adding that, however, a minimally invasive orthodontic treatment, such as the use of a removable appliance, could be helpful to promote mental stability and improve occlusion. In their case series, Shelef et al. analyze the use of short-term chloral hydrate as an add-on treatment, reporting how it can improve sleep and relieve agitation in patients with treatment-resistant Schizophrenia (TRS). The authors state that chloral hydrate is effective in showing some short-term benefits in improving sleep disorders and reducing violent and agitated behavior in patients with TRS, providing an optimal alternative in the short-term treatment of Schizophrenia. Delving deeper into the discussion regarding rare conditions in their clinical manifestations, Huang describes a case of rare variants in the *MTRR* gene, *66GG* and *524TT*, which cause hyperhomocysteinemia and folic acid deficiency associated with schizophrenia; the authors describe a case of early-onset psychosis with hypertension and established refractoriness to antipsychotics and antihypertensive drugs. The authors hypothesized that some patients with schizophrenia with abnormal levels of homocysteine or vitamins in the blood may respond to additional vitamin supplementation, as suggested by some studies. Long-term clinical history revealed a case with limited response to pharmacological treatment, with the onset of epileptic events, subsequently highlighting two pathogenic variants (*66GG* and *524TT*) in the *MTRR* gene that caused high levels of homocysteine and low levels of folic acid in the blood. The patient’s serum homocysteine and folate levels returned to normal with vitamin supplementation, although the psychosis did not improve significantly, suggesting the importance of careful clinical monitoring for associated signs. A variant concerning the surgical aspects of the management of the biological bases of Schizophrenia is described by Wu et al. in their report concerning a case of mental disorder caused by shunt blockage after surgery for hydrocephalus. The authors describe how psychiatric symptoms are a significant aspect of the clinical presentation of organic brain lesions and can manifest themselves before the anomalies are detectable through imaging; the evaluation of this case allows for an increased awareness of the multiple aspects involved in such complex conditions, pushing for an improvement in diagnostic accuracy. Furthermore, Fu et al. analyze a case of Electroconvulsive Therapy (ECT)-induced primary open angle glaucoma in a patient with unstable thyroid function; in their report, the authors emphasize that although ECT has been used in patients with coexisting psychiatric and thyroid dysfunctions, there are no reports addressing the risk of inducing or exacerbating glaucoma in the context of unstable thyroid function. This case emphasizes the need to monitor intraocular pressure in patients with unstable thyroid function during ECT to mitigate the risk of ocular complications.

The importance of rigorous methodological standards in case reporting is emphasized by the impact of case report descriptions on enriching the literature (39). The rigorous management of complex cases based on standardized and internationally recognized guidelines such as CARE (CAse REport) (40) appears essential to ensure clarity, transparency and replicability. Although the uniqueness of each case is valuable, contextualizing it within existing literature and theoretical frameworks greatly increases its relevance and impact (41). In an era increasingly dominated by big data and meta-analyses, the individual case report remains a cornerstone of clinical knowledge and innovation (42). Through the lens of individual experience, we are reminded that behind every diagnosis, there is a complex, unique human story that deserves our closest attention (43). Moreover, case reports can serve as an incubator for innovation in diagnostics and therapeutics (44). Descriptions of rare constellations of symptoms, treatment-resistant trajectories, or unexpected responses to off-label pharmacological interventions can suggest hypotheses that ultimately lead to formal research studies (45–49). Case reports provide a crucial way to report adverse effects or complications not yet fully characterized in the literature, informing clinical vigilance and risk management (46, 50). The in-depth study of case reports allows for a significant improvement in scientific knowledge and its impact on better clinical management, achieving better outcomes.
